# Supplementary material for: Evidence on Child Nutrition Recommendations and Challenges in Crisis Settings: A Scoping Review
Source: Int J Environ Res Public Health. 2021 Jun 20;18(12):6637. doi: 10.3390/ijerph18126637 (PMC8296440; doi:10.3390/ijerph18126637)
Supplement: Supplementary file 1 [file ijerph-18-06637-s001.zip › Supplementary File S1. Impact of crisis and interventions.pdf]

Supplementary 1. Impact of crisis on nutrition.

Author	Country / Location	Aim	Key Results
Choudhury et al. (1993)	Bangladesh	Determine the effects of biosocial variables on changes in nutritional status of children under 2 affected by flood	1. The flood had a significant negative impact on child nutritional status: proportion of children in villages affected by the flood who were severely malnourished (61% of weight for age) rose significantly from 5 to 11%<br>2. Adverse effect of flood on nutrition and the effect was dependent on sex of child and intake of vitamin A;<br>-Severely malnourished children was significantly greater among those who had not taken vitamin A.<br>-Flood increased the risk of severe malnutrition among boys: Proportion of boys with severe malnutrition increased after the flood greater than for girls.<br>3. Flood increased the risk of severe malnutrition among boys: Proportion of boys with severe malnutrition increased after the flood greater than for girls.<br>4. Children 18 months old were the most affected by the flood.<br>5. Sex of the child, socioeconomic status, mother's education, and sickness during past 2 weeks had a significant effect on severe malnutrition, but they were not dependent on the flood.
Ninno et al. (2005)	Bangladesh	Assess long-term impact of flood on nutrition of children under 5 years and impact of interventions	Children exposed to the flood were systematically smaller than those who were not exposed, and following the flood did not grow more rapidly, therefore did not recover from the calamity (suggesting short-term crises can have lasting consequences).

Author	Country / Location	Aim	Key Results
Belesova et al. (2019)	Low- and middle-income countries	Assess drought as a risk factor for undernutrition in children <5 years of age	1. Variable reports on impact of drought children's nutritional status.<br>-One reported increase in negative effect of drought on children's nutritional status<br>- Two reported limited evidence of negative drought effect on child undernutrition.<br>- Four observed a notable deterioration in children's nutritional status with drought.<br>2. Drought in and of itself is never the sole cause of undernutrition,but triggers undernutrition in populations that are already vulnerable<br>3. There is high and critical levels of child undernutrition in vulnerable settings during droughts, indicating the need for short-term response, and the development of preventative strategies in the long-term.
Chen et al. (2011)	China	Identify changes in nutritional status in children under 5 years	The nutritional status in poorer rural areas fluctuated in response to the economic crisis<br>-Prevalence of stunting in infants under 6 months and 6-12 months old in poorer rural areas increased from 5.7%-9.1% and 6.7%-12.5%<br>-Prevalence of anemia in all age groups increased in poorer rural areas, especially in children under 24 months old.

Author	Country / Location	Aim	Key Results
Nandy et al. (2016)	Ethiopia and Nigeria	Evaluate impact of food price increase on undernutrition in children under 5 years	In Nigeria there was a large increase in prevalence of multiple anthropometric failure between 2008 and 2013.<br>-Statistically significant increases in wasting.<br>-Aggregate levels of undernutrition did not change over the ten years, with a majority of Nigerian children under five being undernourished.<br>- 28% of children's nutritional status affected by crisis.
Ahsanuzzaman et al. (2020)	Bangladesh	Assess impact of cyclone on nutritional status of children under 5 years	1. Nutritional gain of children can be compromised by cyclones with long-term development impacts<br>-Lowered Height for Age Z (HAZ), weight for age (WAZ), and weight for height (WHZ) scores<br>-Increased the likelihood of stunting and being underweight<br>-May hider development of children of the whole generation exposed to the disaster
Cornu et al. (1995)	Congo	Measure change in nutritional status of mothers and children under 6 years	1. The prevalence of low birthweight, the percentage of CED among mothers and the percentage of wasting among children <1 year increased.<br>2. The prevalence of stunting decreased, which may be becauselinear growth retardation take a longer time to appear compare to wasting.<br>3. The prevalence of morbidity among children <1 year old was high but not very different (32% in 1986 versus 39% in 1991.<br>4. Age of child, age of mother, schooling of mother and household characteristics such as number of preschool children, economic level and head of household's occupation influcenced changes in anthropometric status.

Author	Country / Location	Aim	Key Results
Dong et al. (2014)	China	Monitor malnutrition morbidity and anaemic prevalence in children aged 5 - 23 months	1. Poorer short term and long term nutrition outcomes significantly increased two years after the earthquake.<br>-Increase in malnutrition<br>-Decrease of body weight was rapid (underweight prevalence from 0 at three months to 5.9%)<br>-Decrease of length shown by stunting prevalence (6.6% at three months to 10.8%)<br>-Wasting prevalence increased (1.3% at three months to 4.0%)<br>-Anaemic prevalence markedly increased (36.5% to 67.5%); increasing percentage of anaemia was more obvious in girls than boys.<br>2. The child's nutritional status continuously worsened and anaemic prevalence was high in areas affected by the earthquake.
Gaire et al. (2006)	Nepal	Assess association between disaster and stunting in children aged 5- 59 months	1. Prevalence of stunting is 43% (17.1% severely and 25.9% moderately).<br>2. Child stunting is associated with floods (adjusted OR =0.42, 95% CI: 0.26, 0.67 and adjusted OR =0.59, 95% CI: 0.42,0.83 for severe and moderate stunting, respectively).<br>3. Children aged 6–11 months (adjusted OR =0.26; 95% CI: 0.13, 0.52; P=0.000) were significantly more likely to be moderatly stunted than the older children.<br>4. Children aged 36–47 months (adjusted OR =0.11; 95% CI: 0.03, 0.33; P=0.000) were significantly more likely to be severely stunted than the younger children.
Martin-Prével et al. (2001)	Congo	Examine the role of routine health activity attendence and changes in nutritional situation among children aged 4-23 months	1. Dramatic decline in routine health services received in worsening socioeconomic conditions.<br>2. Attendence of routine health services health activities index was also strongly linked to the nutritional status of the children.<br>-Higher decrease in nutritional status (height for age) with decrease in attendence of routine health service, showing the importance and mediating effect of routine health activities on nutriton of children.
Mulder-Sibanda (1998)	Haitai	Identify nutritional status in children aged 6–59 months	1.Between 1976 and 1980, gross domestic product (GDP) grew more than 4% annually and prevalence of stunting, underweight, and wasting in children fell considerably.<br>2. 1991–1994, political instability and an international trade embargo took a toll on GDP growth, causing it to decline dramatically causing rates of stunting and underweight to level off, while wasting nearly doubled<br>-Child nutrition is dramatically affected during periods of intense political crisis

Author	Country / Location	Aim	Key Results
Nidzvetska et al. (2017)	Ukraine	Explore perceived health, barriers to access to healthcare, caring practices, food security, and overall financial situation of mothers and young children under 2 years	1. Perceived physical health of mothers and their children was found not to be affected by conflict and displacement.<br>2. Not identified malnutrition, but rather found unbalanced nutrition practices, leading to negative health effects of which tend to appear later.<br>3. Most were well-informed about breastfeeding duration and health benefits it brings to a child and reported no breastfeeding disruptions due to their conflict and displacement experience.<br>4. Significant changes in diet after displacement were reported.
Pongou et al. (2006)	Cameroon	Assess factors associated with nutritional status in children under 3 years	1. Over the period of the severe economic crisis average weight-for-age z-score and height-for-age z-score declined respectively from -0.70 SD to -0.83 SD (p = 0.006) and from -1.03 SD to -1.14 SD (p = 0.026)<br>2. Special attention should be paid to the possible role of breastfeeding in early ages (children aged 0–5 months), as it has been proven to have great benefits to child health and survival.<br>-Economic status had little effect in children aged 0–5 months, but significantly positive effect in older ages, which may be attributed to the due to the role of breastfeeding.<br>3. Maternal education and maternal health seeking behavior were associated with better child nutrition.
Rajmil et al. (2014)	Global	Provide an overview of the impacts of crisis on the health of children under 18 years	1. Most studies suggest that the economic crisis has harmed children's health, and disproportionately affected the most vulnerable groups.<br>2. Mortality: excess of 28,000–50,000 infant deaths in 2009 was estimated in sub-Saharan African countries, and increased infant mortality in Greece.<br>3. Studies in Spain, UK, U.S. and Bangladesh all demonstrated significant adverse effect of the economic crisis on food intake by children.
Nielsen et al. (2004)	Guinea-Bissau	Evaluate the effect of an supplementary feeding programs on malnourished children	1. Prevalence of malnutrition increased with the start of the war but then decreased.<br>2. Mortality of malnourished children did not increase during the war<br>2. 67% treated recovered from malnutrition

Supplementary 2. Child nutrition interventions and their impact in crisis.

Author	Aim	Intervention Type	Intervention Detail	Key Results
Kurdi et al. (2020)	Reduce prevalence of child malnutrition through increasing womens knowledge on child nutrition	1. Cash for Nutrition<br>2. Nutrition education<br>3. Malnutrition screening	1. The manual and visual materials were developed by the Ministry of Population and Health on the basis of messages from UNICEF and WHO localized to the Yemeni context<br>2. Monthly cash transfers of 10,000 Yemeni riyals (25% of the value of average monthly food spending) conditional on attendance at monthly nutritional training sessions led by locally recruited community health volunteers.<br>3. 6 days of training in general health education and 6 days of training in nutrition education: Early breast feeding until 6 months, complementary feeding from 6 to 24 months, nutritious meals, handwashing, treatment of drinking water, how to treat diarrhoea, breastfeeding initiation and the importance of colostrum as well as the consequences of chewing qat and smoking during pregnancy.<br>4. Home visits by community health volunteers to detect and refer cases of malnutrition to health centers for treatment.	1. Cash for Nutrition programme was effective in improving women's knowledge and practices.<br>- Significant increase in the probability of reporting breastfeeding initiation within the first hour after delivery for children under two at the time of survey (15.6% increase).<br>- Significant impact on the probability of EBF for women with children under 6 months (15.6% increase).<br>- Significant impact on the share of households that treated drinking water for children under two (10.3%).<br>- Strong positive time trend on the water treatment outcomes, particularly for treatment of water for children under two (16% increase).<br>- Increased correct answers on breastfeeding initiation (17.7%).<br>- No significant impact but positive time trend showing an increase in correct answers on EBF (9.3% point).<br>2. Success in trainings being conducted by local community health volunteers to build trusting relationships and no need for strict administrative oversight.<br>3.Positive trend in probability of treating water and breastfeeding initiation for the control group, showing that outcomes improved even for nonparticipants in the programme who were living in communities where other women were participating.

Moench-Pfanner et al. (2005)	Free up household cash to permit increased consumption of micronutrient-rich foods to reduce the prevalence of irondeficiency anemia	1. Food for Work<br>2. Supplementary feeding program	1. Work: Rehabilitation of community infrastructure, Skills training for urban livelihood (i.e., craft-making cooperatives), Maintenance of community infrastructure and sanitation, Health and nutrition education, and Improved agriculture and fishing techniques.<br>2. Food: Rice, oil, pinto beans.	1. No effect of the FFW programs on reducing child (24–59 mo) and maternal anemia, except in Surabaya, where program participation prevented an increase in maternal anemia<br>2. Freed-up household resources were used to cover a wide range of costs, and hence did not increase micronutrient intake as much as needed.
Balaluka et al. (2012)	Raise mothers' awareness of the benefits of breastfeeding and the need to practice exclusive breastfeeding from birth for a period of six months.	Nutrition education	1. Program developed in South Kivu by the Ministry of Public Health and the National Nutrition Program (PRONANUT)<br>2. Training to prepare for birth and practice exclusive breastfeeding for 6 months.<br>3. Door to door visits and community meetings by community health workers to promote exclusive breastfeeding	1. Community volunteers can improve the utilization of primary health services<br>-Higher skilled birth attendance (93% compared to 38%)<br>2. Improvement in breast feeding<br>-Significantly longer length of exclusive breastfeeding<br>-Higher proportion of exclusively breastfed from birth to 6 months (92% compared to 51%)<br>3. Improvement in health outcomes<br>-Reduced child mortality<br>-Higher weight of infants at 12 months (8.42 kg comparedto 7.97kg)
Giles et al. (2014)	Protecting child nutritional status following financial crisis	Supplementary feeding program	1. Infants (6-12 months): Soft meals (360–430 kcal of energy per 100 g of food and 10-15g of protein). Consumed over three to four feedings during the day for 180 consecutive days.<br>2. Young toddlers (12-24 months): 90 food supplements (360–430 kcal with 9-11 g protein per day) for over 12 months.<br>3. Children (24-60 months): 90 locally prepared snacks<br>(360–430 kcal and 9-11 g of protein).	1. Significant improvement on moderate stunting in children 12-24 months<br>-More significant improvement in boys<br>2. Significant improvement on severe stunting forchildren 6 to 60 months<br>-87 children under 5 years improved from extreme stunting to stunting<br>-18 children under 2 years improved from extreme stunting to stunting<br>3. Program was expensive and the study was not able to assess efficiency of intervention in reducing malnutrition.

Choudhury et al. (1993)	Determine the effects of biosocial variables on changes in nutritional status of children under 2 affected by flood	Supplementary feeding program	Vitamin A	Vitamin A protected children from severe malnutrition, indicating a need to distribute vitamin A to children in areas susceptible to natural disasters to reduce the likelihood of severe malnutrition.
Ninno et al. (2005)	Assess long-term impact of flood on nutrition of children under 5 years and impact of interventions	Supplementary feeding program	Distribution of rice	1. Programs of assistance designed to intervene after the crisis are ineffective compared to programs designed to maintain children's health over the long run.<br>2. Government assistance is often poorly targeted to flood-exposed households.<br>-Gratuitous Relief (GR), such as short-term relief through distribution of small quantities of rice designed to provide emergency relief to disaster victims, explicitly targeted flood-exposed households, but assistance also went to non-flood households.<br>-Vulnerable Group Feeding (VGF), such as transfer of rice per month, aimed at assisting households over a longer period, did not explicitly target flood-exposed households.<br>3. Governments can help to minimize and alleviate the adverse consequences of a disaster by providing assistance to the most vulnerable.

Belesova et al. (2019)	Assess drought as a risk factor for undernutrition in children <5 years of age	Overall		1. Four studies mentioned nutritional programmes existing in their study areas prior to the drought, and twelve interventions implemented in response to the drought.<br>2. Variable effectiveness of the interventions inmitigating drought impacts on children's nutritional status.<br>-One reported decline in child underweight levels during a drought six months after the implementation of an intervention<br>-Two suggested that there was no difference in the levels of child undernutrition between the areas with and without the interventions.<br>3. Interventions that address the underlying vulnerabilities could have high value in strengthening the resilience to the impacts of drought and leveraging overall improvements in child nutritional status.
Chen et al. (2011)	Identify changes in nutritional status in children under 5 years	Nutrition policy		Nutritional status of children under 5 was comparatively stable during and after the global economic crisis, attributable to the Chinese government's policy response.<br>-Prevalence of underweight and stunting in children under 5 had a downward trend<br>-Underweight prevalence was close to normal

Nandy et al. (2016)	Evaluate impact of food price increase on undernutrition in children under 5 years	Nutrition policy	Food Security Programme	1.Ethiopia has a policy climate and effective child nutrition policies (such as the Food Security Programme) which resulted in an impressive improvement in child nutrition.<br>-Impressive declines in multiple anthropometric failure.<br>-Statistically significant declines in the prevalence of both stunting and underweight<br>-Significant decline in prevalence of wasting only between 2005 and 2011, which is impressive considering this was the period when food prices increased most.<br>2. Nigeria has no similar policies on child nutrition<br>-Little or no change was apparent in nutrition outcomes.<br>-Relatively small decrease observed for stunting, between 2008 and 2013.<br>-Large increase in prevalence of multiple anthropometric failure between 2008 and 2013.<br>3. Protectionist public health and nutrition interventions can mitigate the impacts of price increases on poor children.
Balhara et al. (2017)	Identify and describe the effect of nutrition interventions in disaster settings for children under 18 years	Overall		1. Nutrition interventions have the potential to decrease morbidity and save lives in disaster and emergency settings.<br>2. While the WHO 2006 standards appear to have been widely adopted,there is a need for predetermined consensus outcomes in future nutrition supplementation studies.<br>3. As regions of conflicts and displaced people continue to evolve to include new areas and populations, future research on nutrition interventions must evolve to include those populations.

Lopriore et al. (2004)	Assess the effect of a highly nutrient-dense spread fortified with vitamins and minerals in correcting retarded linear growth and in reducing anemia in stunted refugee children	Supplementary feeding program	Micronutrient fortified spread (50g/d fat spread + vitamin-mineral mix fortification) daily +/- Metronidazole (250mg BID x 5 d)	1. Fortified spread induces catch-up growth in stunted children whose diets are poor in micronutrients.<br>-Linear growth of children fed fortified spread was 30% faster at 3 months<br>-Height-for-age z scores increased<br>-Increase in hemoglobin concentrations at 6 months<br>-Anemia was reduced by nearly 90%
Kumar et al. (2005)	Results from a case study of managing child malnutrition in drought affected areas	1. Supplementary feeding program.<br>2. Nutrition education	1. Nutrition care centers<br>2.Targeted feeding (3 times in-center and 2 times take home)<br>3. Nutrition and health education (focus on breastfeeding up to six months and complimentary feeding after six months, and feeding of children during illness, and of iodized salt)	1. Reduction in prevalence of under nutrition in children from 66.7% to 59.6%.
Dong et al. (2013)	Assess effectiveness of complementary food supplements	Supplementary feeding program	1. Study was supported by UNICEF with the nutrition quality and safety evaluated and monitored by NationalInstitute of Nutrition and Food Safety Chinese Center for Disease Control and Prevention (NINFS-CCDC) designed by UNICEF.<br>2. Daily supplement of Ying-Yang Bao (protein 3.0 g from soybean, vitamin A 250 micrograms, vitamin D3 200 IU, vitamin B1 0.3mg, vitamin B2 0.3mg, iraon 5 mg,<br>zinc 5mg, calcium 250 mg; 40kcal energy, 2g fat, 2.5 g carbohydrates)	1. Decrease in antomometric failures<br>-Wasting (WLZ<-2) (3.5% to 1.7%)<br>-Stunting (LAZ<-2) (8.9% to 5.0%)<br>-Underweight (WAZ <-2)(4.5% to 3.3%)<br>2. Reduced anemia prevalence (74.3% to 37.4%)
Hossain et al. (2009)	Assess relationship between food aid and acute malnutrition among children	Supplementary feeding program	Food aid	1. Children in households receiving food aid had a lower prevalence of acute malnutrition but results were not statistically significant<br>-Decrease in underweight (WAZ <-2) adjusted OR

				0.40 95% CI 0.13-1.22 and adjusted OR 0.72 95% CI 0.36-1.44 in two study areas
Ndemwa et al. (2011)	Evaluate the effect of the availability of home fortification with a micronutrient powder	Supplementary feeding program	Daily supplement of MixMe (vitamin A 100 mcg, vitamin D3 5 mcg, vitamin E 5mg, vitamin K 30 mcg, thiamin 0.5mg, riboflavin 0.5mg, pyridoxine 0.5mg, folic acid 90 mcg, niacin 6mg, vitamin B12 0.9 mcg, vitamin C 60mg, iron 2.5mg, zinc 2.5mg, selenium 17mcg, copper 0.34 mcg, iodine 30mcg, maltodextrin) Iron was calculated based upon reference values for medium bioavailability.	1. Micronutrient powder was associated with a small improvement in iron status<br>2. Micronutrient powder led to no significant change in hemoglobin
Mangoni et al.	Evaluate impact of intervention	1. Supplementary feeding program.<br>2. Nutrition education	1. Iron supplementation (for 12,300 children) (Direct iron and vitamin A&D supplementation based on therapeutic or preventive WHO protocols)<br>2.Free treatment for common diseases and food supplementation to vulnerable families (for 3,275 children found anaemic or/and malnourished)<br>3. Community health education	1. Decrease in antomometric failures<br>-Wasting (WLZ<-2) (6.0 to 1.4%)<br>-Underweight (WAZ <-2)(10.9 to 3.8%)<br>2. Decrease in Anemia prevalence (30.1% to 18.8%
Yang et al. (2015)	Explored an ideal way to prevent anemia among children younger than 5 years after disasters	Nutrition education	3-day training courses on health and nutritional education consisting of 9 topics: (1) concepts in food and nutrition; (2) basic nutrition knowledge; (3) when and how to add the complementary food; (4) homemade complementary food; (5) benefits of breast-feeding; (6) preventing common diseases such as upper respiratory infection, diarrhea, and vitamin D deficiency rickets; (7) how to obtain a balance meal; (8) the hazard of iron deficiency and anemia to children as a public health problem; and (9) food iron sources.	1. Improvements in feeding knowledge and practice related to anemia<br>2. Prevalence of anemia decreased from 14.3% to 7.8%<br>3. Hemoglobin concentration increased significantly from 118.8 ± 10.5 to 122.0 ± 9.9 g/L

Rossi et al. (2007)	Evaluate the impact and appropriateness of programmes for the management and treatment of severe malnutrition in emergency situations	Supplementary feeding program	1. Outpatient nutritional centers<br>2. Therapeutic and supplemental feeding	1. Decrease in mortality rate from 6/10,000 to 3.1-4.9/10,000
Grijalva-Eternod et al. (2018).		Cash for Nutrition	1. Monthly unconditional cash transfer of US$84.00 for 5 month (monthly cash amount was based on the cost of the Minimum Expenditure Basket developed by the Food and Agriculture Organization's Food Security and Nutrition Analysis Unit–Somalia.)<br>2. Once-only distribution of a non-food-items kit<br>3. Provision of piped water free of charge.	1. Improved wealth and food security<br>-Increased monthly household expenditure by US$29.60 (95% CI 3.51; 55.68)<br>-Increased the household Food Consumption Score by 14.8 (95% CI 4.83; 24.8)<br>-Decreased the Reduced Coping Strategies Index by 11.6 (95% CI 17.5; 5.96)<br>2. Did not appear to reduce acute malnutrition risk in IDP camp children<br>-Unadjusted hazard ratio 0.83 (95% CI 0.48; 1.42)<br>-Hazard ratio adjusted for age and sex 0.94 (95% CI 0.51; 1.74)
Jayatissa et al. (2012)	Assess the impact of community-based management of acute malnutrition among children	Supplementary feeding program	1. Nutrition Rehabilitation Program,and a manual for health workers on the management of acute malnutrition was prepared, adopting the World Health Organization (WHO) manual.<br>2. Children with severe acute malnutrition were given ready-to-usetherapeutic food.<br>3. Children with moderate acute malnutrition were given 100 g (450 kcal) of high-energy biscuits (HEBs).<br>4. All children received daily supplementary food consisting of locally produced Thriposha or 50 g of corn–soya blend that provides approximately 200 kcal in addition to the general food ration.	1. Decrease in antomometric failures<br>-Wasting (WHZ<-3) (3.5% to 0.7%)<br>-Wasting (WHZ <-2)(18% to 9.6%)<br>2. Increased recovery rates from malnutrition

MirMohamadalile et al. (2019)	Explore the barriers to appropriate lactation after disasters	Nutrition Education		Training programs, health system support, controlling the distribution of breastfeeding materials and providing a supportive environment for breastfeeding can help overcome the breastfeeding barriers in disasters.

Key issues and barriers to nutrition intervention implementation

Author	Country / Location	Key Results
Dong et al. (2014)	China	Lack of feeding knowledge in parents<br>-Only 10% children could have breastfeeding within one hour after delivery<br>-Basic exclusive breastfeeding was low.<br>-Children only ate the same meals as the adults, resulting in poor quantity and quality of complementary food for infants and young children.<br>- More than 90% children never received nutrient supplements.
Akik et al. (2020)	Syria	1. Restricted access was reported by several key informants as one of the challenges to conducting needs assessments and delivering health and nutrition interventions in hard-to-reach areas where the security situation was most severe.<br>2. The health and nutrition responses had to adapt by compromising certain planned interventions or prioritising certain ones over others. Resource limitations were cited to influence, for example, the ability to provide neonatal intensive care.<br>3. The lack of data and evidence on infant and young child feeding (IYCF) indicators contributed to delaying the nutrition response.<br>4. Health and nutrition responses focused on the delivery of the essential, lifesaving, cost-effective primary healthcare packages, such as the Minimum Initial Service Package (MISP), Integrated Management of Childhood Illness (IMCI) which existed previously in Syria since 2000, and nutrition surveillance. However, this modality of delivering healthcare was resisted by Syrian beneficiaries at first due to the reported pre-conflict pattern of by-passing primary services and specialist visits being the first point of contact.<br>5. Response evolves over time, and as priorities were being met there was more opportunity to deliver second- and third-line interventions. For example, within the nutrition sector in Damascus, it was described how once programs for the treatment of acute malnutrition were well-established, organisations were then able to move to supporting the baby-friendly hospital initiative. IDPs might not always be aware of the facilities where they can seek health services.
MirMohamadaliIe et al. (2019)	Iran	1. Factors affecting breastfeeding in disaster situations:<br>- Maternal mental and physical health (such as depression caused by the loss or damage to loved ones in the disaster and lack of proper nutrition by the mother or maternal illness or injury)<br>- Mothers' knowledge: maternal lack of self efficacy and information such as correct method of breastfeeding to feed and care for the child.<br>-Child confusion in identifying the nipple and refrained from sucking the mother's nipple<br>-Cultural belief: lack of a safe and private environment for infants' feeding<br>-Lack of knowledge about breastfeeding among health care staff<br>2. Formula is mostly distributed without any supervision (factories or drug distribution companies donated formula to the relief organizations for the use of people in affected areas only with the purpose of advertisement)
