# Supplementary material for: Evidence on Child Nutrition Recommendations and Challenges in Crisis Settings: A Scoping Review
Source: Int J Environ Res Public Health. 2021 Jun 20;18(12):6637. doi: 10.3390/ijerph18126637 (PMC8296440; doi:10.3390/ijerph18126637)
Supplement: Supplementary file 1 [file ijerph-18-06637-s001.zip › Supplementary File S2. Summary of guidance.pdf]

Supplementary 2. Summary of guidance/ guidelines by issues (Breastfeeding/ Breast-milk substitutes/ Complementary feeding).

Guidance/ Issue	Breastfeeding	Breast-milk substitutes	Complementary feeding
WHO-2004: Guiding principles for feeding infants and young children during emergencies	Principle 1 Infants born into populations affected by emergencies should normally be exclusively breastfed from birth to 6 months of age.<br>1.1 Every effort should be made to identify alternative ways to breastfeed infants whose biological mothers are unavailable.	Principle 3<br>The quantity, distribution and use of breast-milk substitutes at emergency sites should be strictly controlled.<br>3.1 A nutritionally adequate breast-milk substitute should be available, and fed by cup, only to those infants who have to be fed on breast-milk substitutes.<br>3.2 Those responsible for feeding a breast-milk substitute should be adequately informed and equipped to ensure its safe preparation and use.<br>3.3 Feeding a breast-milk substitute to a minority of children should not interfere with protecting and promoting breastfeeding for the majority.<br>3.4 The use of infant-feeding bottles and artificial teats during emergencies should be actively discouraged.	Principle 4<br>To sustain growth, development and health, infants from 6 months onwards and older children need hygienically prepared, and easy-to-eat and digest, foods that nutritionally complement breast milk.<br>Principle 5<br>Caregivers need secure uninterrupted access to appropriate ingredients with which to prepare and feed nutrient-dense foods to older infants and young children.<br>5.1 Adequate feeding of infants and young children cannot be assured if the food and other basic needs of households are unmet.<br>5.2 Blended foods provided as food aid, especially if they are fortified with essential nutrients, can be useful for feeding older infants and young children. However, their provision should not interfere with promoting the use of local ingredients and other donated commodities for preparing suitable complementary foods.<br>5.3 Complementary foods should be prepared and fed frequently, consistent with principles of good hygiene and proper food handling.

Guidance/ Issue	Breastfeeding	Breast-milk substitutes	Complementary feeding
FAO-2005: Resource guide: Protecting and promoting good nutrition in crisis and recovery	In the crisis that may separate family members and kinship ties, the inter-generational transfer of knowledge and skill can lead to inadequate knowledge of breastfeeding and complementary feeding practice. To protect positive breastfeeding practice, the nutrition education interventions should be considered for all mother, fathers, care takers, as well as influential persons in the community and there should be social support and mechanism to address specific constraints. For example, it may be necessary to supply an additional or special ration for lactating women. Mother- and baby-friendly spaces (e.g. breastfeeding stations) can be provided with counselling support from peer mothers.	One compromise would be to give infant formula with preparation instructions in the local language, and no brand name on the label in order to avoid providing free advertising for formula companies. This does not always happen in practice. It is sometimes recommended that no infant formula should be provided.	The design and implementation of interventions to promote improved complementary feeding (from six months of age, in addition to breastfeeding) should take the following issues into account<br>• Complementary food mixes should be based on locally available foods, and should require only small modification to the family diet<br>• Recipes need to be developed and pre-tested with different groups of mothers. Ingredients should reflect the seasonal availability of food, and additional time, water, fuel and other resource requirements should be considered carefully.<br>• Complementary food mixes should be checked for nutrient density. If germination or fermentation techniques already exist, these should be encouraged to improve the digestibility and reduce the bulk of porridges. Theses method also reduce phytic acid level in wholegrain cereal flours, which can inhibit calcium and iron utilization<br>• Feeding centres or group kitchens could be utilized for preparing complementary food mixes as an income generation activity for elderly or partially disabled people<br>• The use of complementary food should be integrated with the promotion of breastfeeding to avoid the substitution of breastmilk in the first year after birth.<br>• If blended food is included in the general ration or through supplementary feeding programmes, practical nutrition information needs to be given and demonstration sessions organized so that mothers can learn how to prepare and feed the blended food appropriately.

Guidance/ Issue	Breastfeeding	Breast-milk substitutes	Complementary feeding
2009-IFE Core Group: Complementary Feeding of Infants and Young Children in Emergencies	Infants and young children are fed in a variety of ways, and feeding practices are the result of complex political, psycho-social, cultural, economic, and commercial influences and interactions. The resulting practice may or may not involve breastfeeding, let alone the recommendation for exclusive breastfeeding for the first 6 months of life or continued breastfeeding for 2 years or beyond		Complementary Feeding in Emergencies (CFE)<br>To help avert such devastating impacts in emergencies, caregivers will need access to adequate amounts of nutritious, appropriate and safe complementary foods. And while there is no reason for CF to start earlier in emergencies, there may be situations where it is preferable to delay it for slightly longer than 6 months; for example, if sanitation is extremely poor and/or there are no quality foods immediately available to meet the most acute nutrient requirements for iron and zinc
2014-European Commission Directorate-General for Humanitarian Aid and Civil Protection: Infant and Young Children Feeding Emergencies: Guidance For Programming	Breastfeeding and infant and young child feeding support should be integrated into services for mothers, infants and young children	Ensuring that humanitarian assistance does not undermine safe IYCF practices with inappropriate interventions. In the emergency situation, BMS is often donated and widely available mothers might choose giving artificial feeds<br><br>Ensure the provisions of the Code are upheld:<br>• Donated or subsidised supplies of breastmilk substitutes are avoided.<br>• Any decision to accept, procure, use or distribute infant formula in an emergency must be made by informed, technical personnel in consultation with the co-ordinating agency, lead technical agencies and governed by strict criteria.<br>• The use of bottles and teats in emergency contexts should be avoided	

Guidance/ Issue	Breastfeeding	Breast-milk substitutes	Complementary feeding
2017-IFE Core Group 2017: The Operational Guidance on Infant and Young Child Feeding in Emergencies		Minimise the risks of artificial feeding<br>1. Develop plans for prevention and management of donations of BMS, other milk products and feeding equipment in an emergency.<br>2. Communicate government position on not seeking or accepting donations to key actors, including country embassies, donors, development partners and civil society groups, among others.<br>3. Use scenarios to forecast potential artificial feeding needs in an emergency-affected population and develop preparedness plans accordingly.<br>4. Establish systems for management of artificial feeding, including coordination authority (or at least terms of reference), BMS supply chain and monitoring mechanisms	
2018-Sphere Association: The Sphere Handbook: Humanitarian Charter and Minimum Standards in Humanitarian Response	Emphasise protecting, supporting and promoting breastfeeding, complementary feeding and hygiene.<br>- Provide clear information on the importance of exclusive breastfeeding in children up to six months, and continued breastfeeding for children from 6 to 24 months, for both the physical and psychological health of mother and child.<br>- Admit breastfeeding mothers of acutely malnourished infants under six months to supplementary feeding programmes, independent of maternal nutrition status		

Guidance/ Issue	Breastfeeding	Breast-milk substitutes	Complementary feeding
2018- WHO and UNICEF: HIV and Infant feeding in emergencies: Operational Guidance	• Mothers living with HIV (and whose infants are HIV uninfected or of unknown HIV status) should exclusively breastfeed their infants for the first 6 months of life, introducing appropriate complementary foods thereafter, and continue breastfeeding for at least 12 months and up to 24 months or longer, while being fully supported for ART adherence. Breastfeeding should then only stop once a nutritionally adequate and safe diet without breast milk can be provided	Principle 7. Where replacement feeding is indicated, supplies of BMS should be based on an individual needs assessment, purchased, and targeted to those in need, with commitment to continued supplies and supportive nutrition, water, sanitation and hygiene, and health care. Where individual-level access is compromised, the designated authority for coordination of IYCF in emergencies should be consulted for advice on adapted options. Donations of BMS should not be sought or accepted in emergencies.<br><br>When mothers known to be living with HIV decide to stop breastfeeding at any time, infants should be provided with safe and adequate replacement feeds to enable normal growth and development.<br>Alternatives to breastfeeding include:<br>• For infants less than 6 months of age:<br>— commercial infant formula milk, as long as the home conditions outlined next are fulfilled;<br>— expressed, heat-treated breast milk.<br>Home-modified animal milk is not recommended as a replacement food in the first 6 months of life	Conditions needed to safely formula feed<br>Mothers known to be living with HIV should only give commercial infant formula milk as a replacement feed to their HIV-uninfected infants or infants who are of unknown HIV status, when all the following specific conditions are met:<br>• safe water and sanitation are assured at the household level and in the community;<br>• the mother or other caregiver can reliably provide sufficient infant formula milk to support normal growth and development of the infant;<br>• the mother or caregiver can prepare the infant formula milk cleanly and frequently enough so that it is safe and carries a low risk of diarrhoea and malnutrition;<br>• the mother or caregiver can, in the first 6 months of life, exclusively give infant formula milk;<br>• the family is supportive of this practice;<br>• the mother or caregiver can access health care that offers comprehensive child health services.

Supplementary 2 Summary of guidance/ guidelines by issues (Supportive environment / Management of Malnutrition)

Guidance/<br>Topic	Supportive environment	Management of Malnutrition
WHO-2004: Guiding principles for feeding infants and young children during emergencies	Principle 2<br>The aim should be to create and sustain an environment that encourages frequent breastfeeding for children up to two years or beyond.<br>Principle 6<br>Because the number of caregivers is often reduced during emergencies as stress levels increase, promoting caregivers' coping capacity is an essential part of fostering good feeding practices for infants and young children.	Principle 8<br>Nutritional status should be continually monitored to identify malnourished children so that their condition can be assessed and treated, and prevented from deteriorating further. Malnutrition's underlying causes should be investigated and corrected.<br>8.1 Special medical care and therapeutic feeding are required to rehabilitate severely malnourished children
FAO-2005: Resource guide: Protecting and promoting good nutrition in crisis and recovery	Relief commodity distribution mechanisms and public facilities (water, latrines) should be designed to be safe for women and to enable access for people with disabilities (location of facility, timing of distributions). Physical structures may be needed to provide woman- and child-friendly spaces<br>Civic awareness campaigns may be needed to provide information on rights, and reporting procedures for confidential referral and accountability if these rights are violated. These types of interventions require training and skills (counselling, psycho-social, protection and legal), which are often overlooked	
2009-IFE Core Group: Complementary Feeding of Infants and Young Children in Emergencies		There is also interest in using lipid-based nutrient supplements (LNS) in emergency settings not just for the treatment of severe acute malnutrition, but for prevention of malnutrition by ensuring a more nutritionally-adequate ration for the most vulnerable groups, including children of CF age
2014-European Commission Directorate-General for Humanitarian Aid and Civil Protection:<br>Infant and Young Children	Considering the specific needs of infants, young children, lactating mothers and carers across all sectors, enabling access to basic services (e.g. shelter, security, food assistance, WASH, health)	

Guidance/<br>Topic	Supportive environment	Management of Malnutrition
Feeding Emergencies:<br>Guidance For Programming		
2017-IFE Core Group 2017: The Operational Guidance on Infant and Young Child Feeding in Emergencies		Micronutrient supplementation<br>For children aged 6-59 months, multiple-micronutrient supplements may be necessary to meet nutrition requirements where fortified foods are not being provided, in conjunction with other interventions to improve complementary foods and feeding practices.<br><br>Vitamin A supplementation is recommended for children aged 6-59 months. For Pregnant and lactating women, iron and folic acid or multiple-micronutrient supplementation should be provided in accordance with the latest guidance.

Guidance/ Topic	Supportive environment	Management of Malnutrition
2018-Sphere Association: The Sphere Handbook: Humanitarian Charter and Minimum Standards in Humanitarian Response	Breastfeeding support: Mothers of infant inpatients need skilled breastfeeding support as part of nutritional rehabilitation and recovery. This is particularly important for children below six months and for mothers with disabilities. Provide sufficient time and resources, such as a designated private breastfeeding area, to target skilled support and enable peer support.<br><br>Emotional support**:**<br>Emotional and physical stimulation through play is important during the rehabilitation period for children with severe acute malnutrition. It promotes attachment and positive maternal mood.<br>Caregivers of such children often require social and psychosocial support to bring their children for treatment. Some mothers may also need to be supported to access mental health care services for perinatal depression. This may be achieved through mobilisation programmes. Programmes should emphasise the importance of stimulation and interaction in treating and preventing future disability and cognitive impairment in children.<br>Enable all caregivers of severely malnourished children to feed and care for their children during treatment; provide them with advice, demonstrations and health and nutrition information. Pay attention to the impact of treatment on the caregivers and siblings to ensure adequate childcare arrangements, avoid family separation, minimise psychosocial distress and maximise the potential treatment adherence.	Breastfeeding mothers of severely malnourished infants under six months should receive a supplementary food ration regardless of their nutritional status. If those mothers meet the anthropometric criteria for severe acute malnutrition, admit them for treatment<br>Management of Moderate acute malnutrition<br>1 Establish clearly defined and agreed strategies, objectives and criteria for set-up and closure of interventions from the outset of the programme.<br>2. Maximise access to coverage of moderate acute malnutrition interventions through community engagement and involvement from the beginning.<br>3. Establish admission and discharge protocols, based on nationally and internationally accepted anthropometric criteria.<br>4. Link the management of moderate acute malnutrition to the management of severe acute malnutrition and to existing health services.<br>5. Provide take-home dry or suitable ready-to-use supplementary food rations unless there is a clear rationale for on-site feeding.<br>6. Emphasise protecting, supporting and promoting breastfeeding, complementary feeding and hygiene.<br><br>Management of Severe acute malnutrition<br>1. Establish clearly defined and agreed strategies, objectives and criteria for set-up and closure of interventions from the outset of the programme.<br>2. Include inpatient care, outpatient care, referral and community mobilisation components in the management of severe acute malnutrition.<br>3. Provide nutrition and healthcare according to nationally and internationally recognised guidelines for the management of severe acute malnutrition.<br>4. Establish discharge criteria that include anthropometric and other indices.<br>5. Investigate and act on causes of default and non-response, or an

Guidance/ Topic	Supportive environment	Management of Malnutrition
		increase in deaths.<br>6. Protect, support and promote breastfeeding, complementary feeding, hygiene promotion, and good mother and child interaction.<br><br><u>Management of Micronutrient deficiencies</u><br>1. Collect information on the pre-crisis situation to determine the most common micronutrient deficiencies.<br>2. Train health staff in identifying and treating micronutrient deficiencies.<br>3. Establish procedures to respond to micronutrient deficiency risks.<br>4. Link micronutrient responses with public health responses to reduce diseases commonly associated with crises, such as vitamin A to manage measles and zinc to manage diarrhoea.

Guidance/ Topic	Supportive environment	Management of Malnutrition
2018- WHO and UNICEF: HIV and Infant feeding in emergencies: Operational Guidance	Principle 4. Interventions should focus on supporting caregivers living with HIV and channeling resources to meet the nutritional needs of the infants and young children in their charge, and to provide or re-establish supplies of ARVs to avoid disruption of treatment. Mothers living with HIV, and their infants, should have their health and nutrition needs prioritized.<br>Principle 5. To minimize the risks of mortality and morbidity related to inappropriate feeding practices, the aim of the emergency response should be to create and sustain an environment that encourages and supports breastfeeding according to international recommendations for children aged up to 2 years or beyond. For infants and young children who have no possibility of breastfeeding, replacement feeding needs to be provided in line with international guidance.<br>Principle 8. Regardless of the infant feeding recommendations for women living with HIV that are promoted during the emergency response, maternal decisions regarding infant feeding should be respected.	

Supplementary 2 Summary of guidance/ guidelines by issues (Assessment & Monitoring / Policy guidance and coordination)

Guidance/<br>Topic	Assessment & Monitoring	Policy guidance and coordination
WHO-2004: Guiding principles for feeding infants and young children during emergencies	<u>Principle 10</u><br>Promoting optimal feeding for infants and young children in emergencies requires a flexible approach based on continual careful monitoring.	<u>Principle 9</u><br>To minimize an emergency's negative impact on feeding practices, interventions should begin immediately. The focus should be on supporting caregivers and channeling scarce resources to meet the nutritional needs of the infants and young children in their charge.
FAO-2005: Resource guide: Protecting and promoting good nutrition in crisis and recovery	Monitoring<br>• Duration of exclusive breastfeeding period<br>• Age at introduction of complementary foods<br>• Increased knowledge and practice of appropriate complementary food preparation<br>• Reduced malnutrition in children receiving complementary foods<br>• Availability of local complementary food products in the market	
2009-IFE Core Group: Complementary Feeding of Infants and Young Children in Emergencies		The challenges posed by emergency situations will make caring for and feeding children harder, and it is therefore crucial to have effective practical guidance for carers, practitioners, and agencies in the field to not only ensure that the nutrition components of CF are met, but help promote, support, and sustain the social and psychosocial elements which may be very much undermined during emergency conditions.

Guidance/<br>Topic	Assessment & Monitoring	Policy guidance and coordination
2014-European Commission Directorate-General for Humanitarian Aid and Civil Protection:<br>Infant and Young Children Feeding Emergencies: Guidance For Programming	Monitoring of interventions should demonstrate how infant and young child feeding needs are being addressed and outcomes improved. Mothers/carers of children under-2 years of age should be consulted to ensure their concerns are considered.<br><br>Key information on the infant and young child feeding situation and needs should be integrated into routine rapid assessment procedures.<br><br>Simple measures should be put in place in all sectors to ensure the needs of mothers/carers, infants and young children are addressed in the early stages of an emergency. Particular attention should be paid to the following points:<br>• Breastfeeding and infant and young child feeding support should be integrated into services for mothers, infants and young children.<br>• Foods suitable to meet the nutrient needs of older infants and young children must be included in the general ration for food assistance dependent populations.	Paying attention to IYCF right at the onset of an emergency (in needs assessment, policy and coordination, in the first responses).<br>Effective coordination should include IYCF-E considerations across sectors in humanitarian response. The nutrition cluster, government or lead agencies, such as UNICEF, have a role to ensure policies for IYCF-E are developed and widely shared.
2017-IFE Core Group 2017: The Operational Guidance on Infant and Young Child Feeding in Emergencies	<u>Assess and monitor</u><br>1. Develop a profile on IYCF practices and maternal and child nutrition to inform early decision making in an emergency.<br>2. Ensure disaggregated data and recent reports are readily accessible.<br>3. Calculate the prevalence of non-breastfed infants less than six months old and at one year and two years old from existing data.<br>4. Prepare key questions to include in early needs assessment. (background information)<br>5. Identify existing and/or potential national/sub-national capacity to undertake IYCF assessment and surveys.<br>6. Support government to develop policies and procedures	<u>Endorse or develop policies</u><br>1.1 Governments and agencies should have up-to-date policies which adequately address all of the following elements in the context of an emergency: protection, promotion and support of breastfeeding; the management of artificial feeding; complementary feeding; the nutrition needs of PLW; compliance with the International Code of Marketing of Breastmilk Substitutes(BMS) and subsequent relevant World Health Assembly (WHA) Resolutions (the Code); prevention and management of donations of BMS; and infant feeding in the context of public health emergencies and infectious disease outbreaks (see Section 9 Definitions for recommended IYCF practices). Additional context-specific provisions may be necessary, such as for refugees or internally displaced persons (IDP). Provisions may exist as a standalone policy and/or may be integrated into other relevant policies. UNICEF and WHO have key

Guidance/ Topic	Assessment & Monitoring	Policy guidance and coordination
	to monitor for and act on Code violations. Monitor and report Code violations to relevant authorities.<br>7. Identify what existing monitoring and evaluation tools and systems can be applied in an emergency context and agree any necessary adaptations.<br><br><u>General</u><br>4.1 Assess the needs and priorities for IFE response and monitor the impact of interventions, humanitarian action and inaction. Prioritise assessment of acute needs and difficulties that expose children to the greatest risk. Gather qualitative and quantitative data in preparedness, early needs assessment and representative surveys. Invest in gathering reliable, accurate, systematic and coordinated information. Triangulate information sources. The level and type of IFE assessment that is possible in a given emergency will depend on a balance of factors including population access, capacity, type of emergency (e.g. acute, chronic) and resources.<br>4.2 Explore opportunities to include IYCF questions in other sector needs assessments and draw on relevant multi-sector data, such as water, sanitation and hygiene (WASH) and health reports. In multi-sector assessment teams, ensure one person has received basic orientation on IFE. For needs assessment planning and analysis, involve personnel experienced in IYCF, ideally IFE. Where such capacity is limited at local level, seek regional and/or global-level specialist support. Consult with sectoral specialists to support analysis of multi-sectoral data as necessary.<br>4.3 Disaggregate data for children under two years old by gender and by age as follows: 0-5 month, 6-11 months, 12-23 months, and proportion of PLW. Informed by the context, disaggregate key information by ethnicity, location,	responsibilities in supporting national/sub-national policy preparedness<br>1.2 In early response, consult national/sub-national preparedness plans, policies and procedures and uphold relevant legislation and international standards. In an emergency, where existing policy guidance is absent, outdated or does not adequately address the context, rapid policy guidance updates or 'stop-gap' guidance development may be necessary, led by the IFE coordination authority and in consultation with WHO, other relevant UN agencies and national/regional/global technical groups.<br>1.3 Develop missing and update existing policy guidance in close collaboration with government authorities and seek to strengthen relevant national/sub-national policies. Develop and update policies and associated procedures in preparedness.<br>1.4 Disseminate key policy guidance to all relevant responders across sectors, including media groups, private sector, donors, military and volunteer groups<br>1.5 An inter-agency joint statement, issued and endorsed by relevant authorities, may be used to highlight relevant guidance, provide context-specific rapid guidance, and harmonise communication. Development of the statement should be led by the IFE coordination authority; UNICEF and WHO have key roles to catalyse and support development. In preparedness, develop a draft joint statement and secure preliminary approval with relevant authorities. A model joint statement is available.<br>1.6 The Code expresses the collective will of governments regarding the marketing of BMS and sets out the responsibilities of the manufacturers and distributors of products covered by the Code, health workers, national governments and concerned organizations. Enact the Code into national legislation in preparedness and enforce at all times, including during emergency response. Ensure that existing legislation is fully in line with the Code. Report Code violations.<br>1.7 Enact legislation and adopt policies in line with the WHO Guidance on Ending the Inappropriate Promotion of Foods for Infants and Young Children). In preparedness,<br>UN, civil society and government policy-makers should develop national

Guidance/ Topic	Assessment & Monitoring	Policy guidance and coordination
	etc. to enable equity analysis.<br><br><u>Pre-crisis data and early needs assessment</u><br>4.4 Use pre-crisis background information (secondary data) to develop an IYCF situation profile to inform early decision-making and immediate actions. Collate key information in preparedness or as necessary, in early response.<br>4.5 Pre-crisis information sources include existing government, NGO and UN country programmes; Multiple Indicator Cluster Surveys (MICS) and Demographic Health Surveys (DHS) etc.<br>4.6 Key information to consider includes: Contexual data (Policy environment, child nutritional status etc.) and IYCF data (Prevalence of breastfeeding or complementary feeding and knowledge and attitudes towards IYCF)<br>4.7 Conduct early needs (rapid) assessment to inform strategic decisions and operational decisions on response. Critical analysis of quantitative and qualitative data is needed to determine appropriate interventions.<br>4.8 Where a representative survey is not feasible, use alternative, opportunistic means to gather relevant data on the current situation. Take account of methodological limitations in analysis.<br>4.9 Gather information for different population groups and develop context-specific indicators as necessary, in consultation with the IFE coordination authority.<br>4.10 Alerts in early needs assessment requiring further investigation include: elevated CMR, IMR and/or U5MR; reports of infant or maternal deaths; global acute malnutrition prevalence above 5%; artificial feeding practiced pre-emergency; low (<50%) exclusive breastfeeding prevalence pre-emergency; mothers reporting difficulties breastfeeding; low (<70%) continued	legally binding policies regarding private sector engagement in emergency response to enable constructive collaboration and avoid undue influence and conflicts of interest.<br><br><u>Protect, promote and support optimal infant and young child feeding with integrated multi-sector interventions</u><br>1. Actively promote and support recommended IYCF practices in the population.<br>2. Integrate the Ten Steps to Successful Breastfeeding of the WHO/UNICEF Baby-friendly Hospital Initiative into maternity services.<br>3. Develop preparedness plans for interventions on breastfeeding support, complementary feeding, artificial feeding and identification and management of particularly vulnerable children.<br>4. Identify key sector focal points in ministries and agencies to engage on programming.<br>5. Profile complementary foods and feeding practices, including existing nutrient gaps and culturally-sensitive response options, and mechanisms for scale-up and response in an emergency context.<br>6. Identify supply chain for an appropriate BMS (if needed) and complementary foods.<br>7. Work to ensure that local/commercially produced complementary foods meet minimum standards.<br>8. Examine national legislation related to food and drugs, particularly importation.<br>9. Anticipate likely need for and mechanisms to provide micronutrient supplementation to PLW and children.<br>10.Develop plans for response and for transition post-emergency regarding IYCF interventions.<br>11.Identify existing or potential public health issues of nutrition concern and plan accordingly.<br><br><u>Coordinate operations</u><br>1. Identify government leadership and coordination authority on IFE and support capacity development to strengthen this responsibility as

Guidance/ Topic	Assessment & Monitoring	Policy guidance and coordination
	breastfeeding prevalence at one year; reports of non-breastfed infants under six months of age; requests for infant formula; poor availability of appropriate complementary foods; infants<br>under six months of age presenting with acute malnutrition; orphaned infants; reports of BMS donations or untargeted distributions of BMS<br>4.11 Where more in-depth assessment is indicated and feasible, conduct a representative survey<br><br><u>Monitoring</u><br>4.12 Intervention strategies should include objectives, target population, expected outputs and outcomes. Define benchmarks to determine progress and achievement considering intervention timeframes.<br>4.13 Use quantitative and qualitative indicators to determine impact of behaviour change activities;<br>4.14 Monitor IFE activities and interventions using standard indicators that are built into monitoring, evaluation, accountability and learning systems where they exist.<br>4.15 Monitor IFE response against higher-level global indicators, e.g. Sphere Standards.<br>4.16 Monitor for Code violations and report them to national authorities. Support government to develop policies and procedures to monitor for and act on Code violations<br>4.17 Ensure that gender equality and equity are integrated consistently in disaster prevention<br>4.18 Use participatory approaches to engage target population groups, including in programme planning and design, feedback sessions and dissemination of findings	necessary.<br>2. Where government capacity is constrained, identify options for coordinated IFE response and leadership.<br>3. Develop terms of reference for IFE coordination in a response.<br>4. Raise public and professional awareness regarding recommended IYCF practices and benefits. Develop an IFE communication strategy and plan for rapid implementation in an emergency.<br><br><u>Prepare easily adapted media briefs.</u><br>5. Engage development agencies and donors in preparedness planning that includes adaptation of existing programmes to meet emergency needs, negotiating funder flexibility to meet new needs and priming sources of surge funding to accommodate increased demands.<br>6. Allocate funding to support monitoring, evaluation and learning.<br>7. Establish links with other sector focal points and coordination mechanisms, especially food security, health and WASH.<br><br><u>Train staff</u><br>2.1 Sensitise relevant personnel across sectors to support IFE, including those dealing directly with affected women and children; those in decision-making positions; those whose operations affect IYCF; those handling any donations; and those mobilising resources for the response. Target groups for sensitisation include government staff, sector/cluster leads, donors, rapid-response personnel, camp managers, communications teams, logisticians, the media, volunteers, among others.<br>2.2 Train personnel on IFE in preparedness and during emergency response, as necessary. Target personnel may include government staff; NGO staff and volunteers delivering health and nutrition services and support at facility or community level; and frontline staff in other sectors.<br>2.3 Adapt and prioritise training content to address identified needs, cultural expectations and personal experiences of mothers and staff; capacity gaps; the target audience; and time available. More specialist

Guidance/ Topic	Assessment & Monitoring	Policy guidance and coordination
		capacity to counsel mothers and infants with heightened needs, such as stressed or traumatised mothers, malnourished infants and mothers, low birth weight (LBW) infants and disabled infants with feeding difficulties, may be needed. At a minimum, staff in contact with mothers and children aged under two years should be trained to be sensitive to psychosocial issues, on nutrition screening, and on referral pathways to more specialist support.<br>2.4 Undertake sensitisation and training in preparedness. Integrate IFE components into existing curricula and trainings and collaborate with national and regional academic and training institutions on content development and delivery. Include basic concepts around IFE and the Code in pre-service training of relevant health professionals. Integrate lessons from previous emergency response into training packages. Document who is trained and how to access them in an emergency.<br>2.5 Identify and use existing national expertise and networks, such as on breastfeeding counselling and support. Sources of national contacts include: Ministry of Health; UNICEF and WHO country offices; World Alliance for Breastfeeding Action (WABA); La Leche Leaguef and other mother-to-mother breastfeeding support organisations; International Lactation Consultant Association (ILCA) and national ILCA affiliates; and International Baby Food Action Network (IBFAN) national groups.
2018-Sphere Association: The Sphere Handbook: Humanitarian Charter and Minimum Standards in Humanitarian Response	Infant and young child feeding assessments: Assess the needs and priorities for IYCF-E and monitor the impact of humanitarian action and inaction on infant and young child feeding practices. Pre-crisis data can be used to inform early decision-making. Work with other sectors to include IYCF-E questions in other sectoral assessments and draw on available multi-sectoral data to inform the assessment Include the number of available breastfeeding counsellors, trained health workers and other support services and their capacity. For more in-depth assessment, conduct random sampling, systematic sampling or cluster sampling. This may be through a stand-alone IYCF-E survey or an integrated survey. However, an integrated survey may result in limited	1. Establish an IYCF-E coordination authority within the crisis coordination mechanism, and ensure collaboration across sectors.<br>• Assume the government is the coordination authority, wherever possible.<br>2. Include the specifications of the Operational Guidance in relevant national and humanitarian organisation policy guidance on preparedness.<br>• Develop guidance and a joint statement with national authorities in situations where there is no policy.<br>• Strengthen relevant national policies wherever possible.<br>3. Support strong, harmonised, timely communication on IYCF-E at all response levels.<br>• Inform humanitarian organisations, donors and media as soon as

Guidance/<br>Topic	Assessment & Monitoring	Policy guidance and coordination
	sample size, which may reduce the representativeness of the survey<br><br><u>Nutrition assessment checklist</u><br>What is the risk of undernutrition related to infant and young child feeding and care practices?<br>• Is there a change in work and social patterns (due to factors such as migration, displacement or armed conflict) affecting the roles and responsibilities in the household?<br>• Is there a change in the normal composition of households? Are there large numbers of separated children?<br>• Has the normal care environment been disrupted (for example, through displacement), affecting access to secondary caregivers, access to foods or<br>access to water?<br>• Are any infants not breastfed? Are there infants who are artificially fed?<br>• Has there been any evidence or suspicion of a decline in infant feeding practices in the crisis? In particular, has there been a decrease in breastfeeding initiation or exclusive breastfeeding rates? Has there been an increase in artificial feeding rates and/or any increase in the proportion of infants not breastfed?<br>• Are age-appropriate, nutritionally adequate, safe complementary foods, and the means to prepare them, hygienically accessible?<br>• Is there any evidence or suspicion of general distribution of breastmilk substitutes such as infant formula, other milk products, bottles and teats, either donated or purchased? In pastoral communities, have the herds been away from young children for long? Has access to milk changed from normal?<br>• Has HIV affected caring practices at household level?	possible about any IYCF-E policies and practices that are in place.<br>• Communicate with affected people about available services, IYCF-E practices and feedback mechanisms.<br>4. Avoid accepting or soliciting donations of breastmilk substitutes, other liquid milk products, feeding bottles and teats.<br>• Donations that do arrive should be managed by the designated authority, in accordance with the Operational Guidance and the Code.<br>• Ensure strict targeting and use, procurement, management and distribution of breastmilk substitutes. This must be based on needs and risk assessment,<br>data analysis and technical guidance.<br><br><u>Infant and young child feeding standard 4.2:</u><br><u>Multi-sectoral support to infant and young child feeding in emergencies</u><br>1. Prioritise pregnant and breastfeeding women for access to food, cash or voucher transfers and other supportive interventions.<br>2. Provide access to skilled breastfeeding counselling for pregnant and breastfeeding mothers.<br>3. Target mothers of all newborns with support for early initiation of exclusive breastfeeding.<br>• Provide simple guidance for exclusive breastfeeding in maternity services.<br>• Protect, promote and support exclusive breastfeeding in infants aged 0–5 months, and continued breastfeeding in children aged six months to two years.<br>• Where mixed feeding is practised in infants aged 0–5 months, support transitioning to exclusive breastfeeding.<br>4. Provide appropriate breastmilk substitutes, feeding equipment and associated support to mothers and caregivers whose infants require artificial feeding.<br>• Explore the safety and viability of relactation and wet nursing where infants are not breastfed by their mother. Consider the cultural context and service<br>availability in such situations.

Guidance/ Topic	Assessment & Monitoring	Policy guidance and coordination
	• Has the general food ration been adapted to the needs of older people and people with difficulties feeding? Evaluate its energy composition and micronutrient content. Assess the acceptability of the food products (palatability, chewability and digestibility).<br><br><u>Food security assessment</u><br>Key Actions<br>1 Collect and analyse information on food security at the initial stage and during the crisis.<br>2 Analyse the impact of food security on the nutritional status of the affected population.<br>3 Identify possible responses that can help to save lives and protect and promote livelihoods.<br>4 Analyse available cooking resources and methods, including the type of stove and fuel and availability of pots and utensils.<br><u>Nutrition assessment</u><br>Key Actions<br>1 Compile pre-crisis information and conduct initial assessments to establish the nature and severity of the nutrition situation.<br>2 Conduct rapid mid upper arm circumference (MUAC) screening and infant and young child feeding in emergencies (IYCF-E) assessments to assess the nutritional situation at the onset of the crisis.<br>3 Identify groups that have the greatest need for nutritional support<br>4 Determine an appropriate response based on an understanding of the context and the emergency	• If breastmilk substitutes are the only acceptable options, include an essential package of support with cooking and feeding equipment, WASH support and<br>access to healthcare services.<br>5. Support timely, safe, adequate and appropriate complementary food support.<br>• Assess household foods to assess whether they are suitable as complementary foods for children and provide context-specific advice and support on<br>complementary feeding.<br>• Ensure access to feeding equipment and cooking supplies, with considerations for children with feeding difficulties.<br>6. Provide feeding support to particularly vulnerable infants and young children.<br>• Support infant stimulation activities and early child development care practices within nutrition programmes.<br>7. Provide micronutrient supplements as necessary.<br>• Provide daily supplements to pregnant and breastfeeding women, including one daily requirement of multiple micronutrients to protect maternal stores and breastmilk content, whether the women receive fortified rations or not.<br>• Continue iron and folic acid supplements when already provided.

Guidance/ Topic	Assessment & Monitoring	Policy guidance and coordination
2018- WHO and UNICEF: HIV and Infant feeding in emergencies: Operational Guidance		Principle 1. Health and nutrition sectors in government and partner agencies must work together on issues related to HIV and infant feeding in emergencies.<br>Principle 3. To minimize an emergency's negative impact on infant feeding practices and ensure nutrition needs are met, preparedness is critical and interventions should begin immediately in the first phase of an emergency response.<br>Principle 9. Preparedness and response need to build on existing systems and national capacity related to HIV and infant feeding.
